# Scientific Advice at a Time of Emergency. SAGE and Covid‐19

**DOI:** 10.1111/1467-923X.12885

**Published:** 2020-08-01

**Authors:** Lawrence Freedman

**Keywords:** science policy, Covid‐19, SAGE, Iraq Inquiry

## Abstract

The challenge for experts in government is often described as one of speaking unwelcome truths to a resistant power. Yet, just as problematic can be instances where the advice is welcome and so left unchallenged. Two such cases in which the UK government followed flawed expert advice are considered: intelligence assessments and military advice leading up to the 2003 Iraq War and the role of SAGE (the Scientific Advisory Group for Emergencies) during the first stages of the Covid‐19 pandemic in 2020. Governments need to interrogate advice and make sure that they understand its underlying assumptions and implications. It remains vital to protect the independence of the experts, but to get the best out of their advice early and active political engagement is required rather than an arms‐length relationship.

when dealing with a crisis, marked by complexity, disruption and uncertainty, governments like to say that they are following expert advice. This seems wiser than relying on intuition or lay advisers. It also provides some cover when things go wrong. But in such situations, where much cannot be known and old models may not apply, experts are likely to differ and offer contradictory opinions. As governments do not like to be put in the position of having to choose between conflicting views, they rely on systems that filter advice and ensure that what reaches senior policy makers is authoritative and represents some sort of consensus. If there is any urgency they cannot wait for the differences to be sorted out at seminars and through peer‐reviewed publications. Yet, a system geared to consensus, even in contentious areas, may mean that what comes through reflects the loudest or most senior voices, with dissenters marginalised and dismissed as mavericks. Those policy makers who worry that they are missing alternative views amongst the expert communities , may look for quite separate sources of advice, if only to enable them to probe and interrogate what is coming through the established channels**.** When consuming expertise it helps to be able to distinguish between what what the experts know with confidence and what is speculation, and to explore the implications of different assumptions. Understanding not only the core messages but also the limits of expert advice is vital when considering its wider political and economic implications when it comes to working out how it best translates into actual policy.

The systems for channelling expert advice to government in the UK have traditionally encouraged the formation of a consensus view before their findings are communicated to senior levels of government. The preferred method is through a committee process. Committees have a number of advantages: they bring individuals with disparate views and responsibilities into the same room and require them to hammer out an agreed position that can then be passed on. This avoids the dangers of being dependent upon a single individual and enables established views to be challenged. It can cope with situations in which the available evidence can legitimately support a number of positions or where judgements cannot be expressed with full confidence. Committees are expected to form an agreed view before their findings are communicated. The main advantage for senior policy makers is that they are not expected to make choices in areas where they lack competence, and can have advice that is sufficiently timely and authoritative to enable them to make decisions. The main disadvantage is that if the science appears to point definitively in one direction, it can narrow down their choices. It is not always apparent that the available evidence might be subject to alternative interpretations with alternative policy implications.

The Covid‐19 pandemic has thrown into sharp relief the importance of the various systems for developing and disseminating scientific advice. In the United States we have seen the government’s best experts trying to hide their dismay as the President offered his own eccentric take on the spread of the virus and what individuals might do to treat themselves. In Sweden the country’s leading epidemiologist encouraged his country to follow a distinctive path, with only modest social distancing measures, which has led to a higher number of deaths than more prudent neighbours, perhaps no more than modest gains in immunity to future infections, but at the same time less economic damage. The German response was helped by a Chancellor who understood the science. In China the science is now being censored to fit in with the approved official narrative of how the country’s responses were both timely and stunningly effective.

Among developed countries the UK has been one of the worst hit by Covid‐19, yet the government has claimed all the way through to have been following the science. By and large, scientists have not contradicted that claim, at least for the period leading into lockdown of March 2020, if not quite so much for the period leading out. From the government’s perspective, the emphasis on following the science carried the implication that this is where an eventual inquiry might usefully start its investigations. For their part, the scientists pointed out that their role was largely to inform policy makers and that policy was bound to take account of many factors beyond the purview of SAGE (the Scientific Advisory Group for Emergencies), the body providing this advice.^1^ A further complication was introduced by reports that the Prime Minister’s Chief of Staff, Dominic Cummings, had sat in on SAGE meetings.^2^ This raised the question of whether the process was being manipulated so that the only advice received fitted with the government’s established preferences.

The allegation put the whole SAGE process under greater scrutiny. Were the selection criteria for participants too narrow? Were important forms of expertise not represented? Why was there so much secrecy surrounding the whole process? A number of scientists, led by a former Chief Scientific Advisor to the government, even set up their own independent source of analysis and advice.^3^ The critics demanded more transparency, unimpressed with the suggestion that names of members should not be disclosed lest they be lobbied. As some members were already in the spotlight because of the importance of their research, and others acknowledged their role, the secrecy was pointless and so the membership of SAGE, along with associated groups, was disclosed. SAGE had already decided on 16 March to place many of the papers that had fed into its discussions onto its website. At the end of May minutes up to its thirty‐fourth meeting on 7 May were published. Thereafter, more was added on a regular basis.^4^ The first batch of minutes showed that Cummings played a minor role (sitting in on four meetings) and that when it came to one of the main criticisms directed against the government—that it was late in imposing lockdown measures—it was indeed following the scientific advice.^5^

There are parallels with the role of the intelligence community in the run‐up to the 2003 Iraq War. The assessments produced by another committee—the Joint Intelligence Committee (JIC)—with regard to Iraqi weapons of mass destruction were not only clearly wrong in retrospect, and could have been suspected of being so in prospect, but were also presented in a published document to provide the justification for the UK policy stance. There were claims that a key government advisor—in this case Alastair Campbell in charge of press communications at Number 10—had influenced the process to get the assessment that suited the government. He was alleged to have ‘sexed up’ the dossier on Iraqi WMD published on 24 September 2002.^6^ The allegation shifted responsibility for the intelligence failure away from the experts and onto the government. The idea that the advice had been manipulated provided a more compelling narrative than the simpler explanation that the original advice was wrong. This idea proved to be extremely durable despite parliamentary committees and three public inquiries finding that it was unfounded.

The Iraq Inquiry, of which I was a member, was able to go into the question of the intelligence assessments and their presentation in some detail. Those doubting its conclusions could look at the extensive evidence published with the resulting Chilcot report, including the original JIC assessments.^7^ The Inquiry noted that not only did the original assessments overstate Iraqi capabilities, but they were not reappraised, even as UN inspectors went into Iraq and failed to find the anticipated evidence of Iraqi WMD. The differences between this case and that of Covid‐19 are more substantial than any similarities. Covid‐19 required bringing together the distinctive inputs from a number of disciplines, notably medicine, epidemiology, and behavioural science, in a fast‐moving situation marked at first by information scarcity and then by overload. The JIC assessments on Iraq focussed hard on one particular question with sparse information. Yet, they are both interesting in one key respect. It is natural for studies on specialist advice to government to focus on those points at which the advisors challenged some fundamental premise of official policy—what might be called ‘truth to power’ moments. In these cases the issue was one of how governments should deal with compelling advice that happened to be convenient, because it fitted their policy predilections, yet had been forged through uncertainty and turned out to be flawed.

In this article I first examine the case of intelligence assessments leading up to the Iraq War. I will also touch briefly on the question of military advice. This is different again because it always contains an operational aspect, as those providing the advice must implement the resulting policy. The Iraq Inquiry pointed to the need to institutionalise forms of challenge into the formation of both intelligence assessments and military advice to prevent groupthink but **also** emphasised that challenges should come from senior ministers and officials. I will then return to the Covid‐19 case and note how the way that problems are framed can have important policy implications. The SAGE system is capable of internal challenge and can respond with alacrity to new evidence. It would, however, have benefitted from more rather than less political engagement during the critical early stages of the pandemic. There is a natural view that the best advice is developed separately from policy discussions so that what reaches ministers has a pure, evidence‐based quality. But it may only be through active discussions with policy makers that the right questions can be identified, unstated assumptions exposed, and the answers presented in such a way that they can be understood and acted upon.

## Intelligence assessments and military advice in the run up to 2003 Iraq War

In 2002 the JIC was based in the Cabinet Office and included representatives of the intelligence agencies (MI5, Secret Intelligence Service (SIS), GCHQ and Defence Intelligence Staff (DIS)) as well as the Home Office, Foreign and Commonwealth Office, Department for International Development, and the Cabinet Office. Until 1998 it had good information with which to assess Iraqi capabilities. Under UN Security Resolution 687 of April 1991, following Kuwait’s liberation from Iraqi occupation, Iraq was obliged to dismantle all of its weapons of mass destruction and to demonstrate to the satisfaction of UN inspectors that it was doing so. By 1998 much had been achieved but there were residual uncertainties about whether some chemical and biological weapons (CBW) capabilities had been hidden, especially as President Saddam Hussein was denying inspectors access to likely sites. The UK and US moved to military action in December 1998 with Operation Desert Fox to pressure Iraq to allow access and to degrade capabilities if it failed to do so. The UN inspectors left Iraq before this operation began and were not allowed to return. Because Iraq had shown an interest in acquiring WMD and readiness to use them prior to the 1991 war, and then continued to attempt to thwart inspections, it seemed reasonable to assume that it would take advantage of the absence of inspections to revive at least chemical weapons capabilities and possibly biological as well. There was solid evidence by 2002 that Iraq was working on missiles, which would make sense as there was little point in developing new weapons unless it had means of delivery. Beyond that the evidence was ‘sporadic and patchy’ and increasingly depended on human intelligence sources that were taken far more seriously than they should have been.^8^

The ministerial consumers of the JIC assessments could not have concluded other than Iraq probably still had some CBW holdings left over from 1991 and was developing new capabilities. UK policy in the months leading up to the war makes no sense unless this is what they believed. This explains the demands for Iraq to address the concerns of the Security Council and allow inspectors to return, at risk of war, which would have been an odd thing to do if it was known for certain that nothing much would be found. Saddam’s continual refusal to comply further convinced the UK and US that they were right and the status of the assessments was elevated. When Iraq agreed to let inspectors back in this was assumed to be a result of their coercive pressure, which it was. The JIC then warned that little might be found, not because there was little to be found, but because Iraq was known to be adept at deceiving inspectors and hiding capabilities.^9^ This meant that JIC had a ready explanation when, as events turned out, the inspectors failed to find a ‘smoking gun’, although they did uncover Iraqi missile programmes in breach of Resolution 687.

Why was the intelligence so wrong? The Chilcot Inquiry argued that the problem began with an embedded construct about Iraqi intentions and capabilities which affected how new information was treated. This was related to the familiar phenomenon of ‘groupthink’ which reduced the readiness to challenge the construct. This was not simply a question of a shared worldview but also the balance of power within the intelligence community. The head of SIS (Sir Richard Dearlove) was invested in the construct because it was his sources that supported it. Even as the sources were disavowed he was reluctant to accept that the construct was wrong. The only challenge came from within the DIS, and this was more about the degree of confidence shown in the assessment rather than its broad thrust. Their concerns were put to the head of DIS, who sat on JIC, as the September 2002 dossier, which was to present the findings to the public, was being finalised. He did not consider them of sufficient merit to reopen debates at such a late stage when a draft document had been agreed in JIC.

The dossier was the result of demands that the government explain the reason for its tough stance on Iraq. This did not lead to JIC producing a new and different assessment, but it was presented with more confidence than the evidence warranted. This confidence was further reinforced by the Prime Minister’s foreword which claimed that it had made the case ‘beyond doubt’.^10^ Once it had gone public with such a firm assessment, the government continued to defend it even when the inspections provided little confirmation. Once inspections had begun, without much being found, the chair of JIC confirmed his confidence in the assessments, including on the eve of the invasion.^11^

At no point did senior policy makers question the assessment, even when they were queried by Dr Hans Blix, in charge of the UN inspections.^12^ At most, they asked for assurances of its continuing validity. As they did not see the raw intelligence they had no basis with which to form an alternative assessment of their own. The only way they shaped the assessments was in how they posed questions to JIC. In this case, in July 2002 JIC was asked how might CBW be used by Iraq in the event of war and then in September how might the evidence on CBW be turned into a document for publication. Both questions took for granted the existence of Iraqi CBW capabilities. Given a strategy based on a demand that UN inspectors return to Iraq, it would have made sense to be sure about what they might find. So an alternative question would have been to ask about the hard evidence behind the assessments and how it might be exposed by inspectors.

Over the same period, the consistent military advice was that the UK should make the largest possible contribution to the US invasion force, including substantial ground forces.^13^ This was despite the readiness of both the Prime Minister and the Secretary of Defence to offer a more modest contribution to US Central Command, responsible for planning the invasion. The reasoning was often tendentious, emphasising the damage that would be done to relations with the US by withholding this contribution, and the influence the UK would gain if it was made, including the possibility that it would enable the UK to commit to the main combat phase of the war while leaving it to others to cope with the aftermath. Although it was apparent that American preparations for the aftermath were poor, at no point did the military suggest that this was a reason not to participate. Some of the Prime Minister’s advisers were sceptical, with one noting a tendency to ‘ventriloquise’ the Americans.^14^ They encouraged the Prime Minister to ask hard questions about the military advice but he chose not to do so

The remit of the Chief of Joint Operations (CJO) is to produce ‘policy aware military advice’.^15^ This is a change from the previous formulation of ‘politically aware’ advice, which might have just meant savvy advice, in which those aspects that might appeal to ministers were highlighted. In principle, the starting point for policy awareness should be the objectives set by government to guide national strategy. It should also require clearly explaining the risks associated with alternative courses of action as well as avoiding options that are not realistically going to be adopted. The challenge for the 'policy aware' advisor is to anticipate the concerns of officials and ministers rather than wait for active engagement.

The Chilcot Report recommended that those who wished to challenge advice from within the intelligence community and the military establishment, including the Ministry of Defence, should be encouraged to do so. This has been incorporated into Ministry of Defence guidelines for giving future operational advice which lists the hard questions that should always be asked before proposing military action.^16^ Devices such as ‘red teams’ charged with demonstrating the weaknesses in proposed actions are one means of exploring the limitations of any advice. The need for challenge may not because the advice is too bold or far‐reaching in its implications. It might have been watered down or compromised to get a consensus when something sharper might have been better. This again points to the importance of not only lower level challenges, but high level interrogation, framing the questions with care and scrutinising the answers critically. This, as Chilcot also noted, can be done most effectively by senior ministers, either at Cabinet or more likely in a Cabinet‐level committee, ideally including ministers without relevant departmental responsibilities, but sufficient experience and weight to turn a sceptical eye to the advice coming forward.

## Move to lockdown February to March 2020

Participants in SAGE include those in key expert positions in government, in this case the science advisors to individual departments and as well as the government’s Chief Scientific Advisor (Sir Patrick Vallance) who acts as chair. In the Covid‐19 case the Chief Medical Officer (Chris Whitty) acted as co‐chair. Other members are key figures from the NHS and Public Health England. But there were independent voices also, including leading academics, some of whom had participated in SAGE’s work on previous epidemics. Feeding into SAGE’s work were sub‐committees, including NERVTAG, and SPI‐M (the modellers) and SPI‐B (the behavioural scientists). These were much more weighted towards academic specialists. It was Vallance and Whitty who had the responsibility for bringing the advice to government, normally by attending COBR, and who provided the main public face of the scientific advice.^17^ Over time the group grew, with more sub‐committees and sub‐sub committees, than it did when the crisis began. By mid‐May, with attendance made easier by Zoom, over fifty people were routinely present at meetings, up from ten to twenty. It now has a large staff to help filter requests from individuals. By contrast with the full information on SAGE and its deliberations, there is not so much on the government’s main decision‐making body, COBR, including how it interacts with the full Cabinet.

By necessity SAGE can be said to provide ‘policy aware scientific advice’. This can be seen in the objectives it set for itself and the means chosen to meet those objectives. The key aim was to prevent the NHS being overwhelmed by a surge of acute cases. SAGE focussed on the demand side of this equation, which meant measures to protect vulnerable individuals as well as interventions to alter the curve of infections using enforced social distancing. Before considering these interventions, it is important to note that addressing the supply side had large consequences, especially in less essential treatments so that wards could be cleared and extra ICU capacity built. It is now known that elderly patients were moved back into care homes, often taking the coronavirus with them. It does not seem that SAGE was asked to advise on the possible consequences of these moves for the transmission of the disease or the associated death toll, or indeed the wider mental health or social costs. SAGE was certainly aware of the care homes issue but assumed that this was being addressed by the responsible authorities. Commenting on the fact that half the deaths appeared to have occurred in care homes, Professor Neil Ferguson of Imperial College and, at this time a member of SAGE, observed : ‘We have always worked under the assumption, which was Government policy at the time, that care homes would be shielded from infection.’^18^

Ferguson also said at the same parliamentary hearings that new analysis suggested that the number of deaths could have been halved had the UK moved to lockdown a week earlier.^19^ SAGE, including Ferguson, was not advocating this at the time so it is instructive to ask why this was not the case when other countries did act earlier than the UK. The SAGE minutes for 27 February contained a ‘reasonable worst case scenario’ in which ‘80% of the UK population became infected with an overall 1% fatality rate’. So this implied (as a worst case) that over 50 million people could get infected with about 500,000 dying.^20^ These were never presented as predictions, but only scenarios on certain assumptions. Nonetheless, they were numbers that were hard to ignore. At issue was how long the UK had before it needed to introduce measures to deal with such a large number of cases. The answer of a few weeks turned out to be optimistic, as people returning from half‐term breaks in Italy and Spain in late February and early March returned with infections and seeded a number of outbreaks across the UK. There was then a delay while the data caught up with a surge in cases. This problem was aggravated by limited testing capacity. So SAGE waited for the data, which was produced in the middle of the month in a dramatic fashion by a study from Imperial College, which demonstrated that the UK was further along the curve than assumed and that without timely action the NHS would not be able to cope. On 16 March stringent measures began to be introduced culminating with the full lockdown on 23 March.

This was almost a month after it had first been suggested, on 25 February, by SAGE that this might be an effective response to the virus. Despite previous scepticism, it was then noted that the evidence from Hong Kong, Wuhan, and Singapore suggested that such measures (‘university and school closures, home isolation, household quarantine and social distancing’) could get the critical reproduction number (the R number) to approximately 1.^21^ A similar effect could be achieved in the UK, helping to slow the epidemic even if it could not be halted.^22^ This led to a paper prepared by SPI‐M for 26 February that was discussed at the next day’s meeting. It included an observation describing whether ‘it is preferable to enact stricter measures at first, lifting them gradually as required, or to start with fewer measures and add further measures as required’ as a 'political decision'. Yet SAGE ended up pushing the second option. This was despite the minutes for 27 February noting that modelling suggested that ‘earlier and/or combined interventions will have more significant impact’.^23^ As a result of this, SPI‐M was asked to ‘develop illustrative scenarios showing the plausible impact of combinations of interventions’ which led to this paper detailing all the options.^24^ The advice resulting from this paper argued for a graduated approach, with interventions to be adopted sequentially. This meant that SAGE was taking what had been described previously as an essentially political position. There were scientific grounds for this approach, as it would allow for the effect of each intervention to be evaluated, but the case was made more on expected levels of compliance with stringent measures and their potential durability.

The discussion at the SAGE meetings on 3 and 5 March showed appreciation that interventions should start, but caution when it came to banning large gatherings and school closures. The issue with lockdown was whether it would be followed sufficiently and sustained for enough time. A new iteration of the paper dated 9 March was tabled on 10 March.^25^ This led to the start of a shift in thinking. SAGE now noted that ‘a tiered approach to social distancing might reduce its impact on the epidemic curve and on mortality’. In theory, the group noted, ‘maximum efficacy from all interventions would be achieved through simultaneous introduction’.^26^ A week later there was seen to be little choice. Infections were going up and the models now demonstrated the risk of intensive care units being overwhelmed. Leading politicians such as former Health Secretary, Jeremy Hunt, were urging quicker action.

## Conclusion

With Iraq, the Chilcot Inquiry established that the problem was not, as is still commonly supposed, that Tony Blair’s government manipulated the system to get the advice that suited their established policy preferences, but that it was prepared to accept superficially uncontroversial advice without scrutinising it. Claims that they were dutifully following intelligence assessments and military recommendations almost de‐politicised their choices. A more careful interrogation of the advice would have helped the government avoid the consequences of an ill‐considered move into war. In the end, the politicians are accountable for policy failures and it will not help them to say that they passively followed the experts.

A model of civil–military relations whereby the military accept whatever political objectives are set by the civilians, but then are left alone to decide on the operations necessary to achieve these objectives, is now accepted as unrealistic. In practice the civilians need to know what objectives are feasible before they are set, and they need to understand the implications of the military means being adopted, as these are rarely politically neutral. This is obviously the case with counterinsurgency and counterterrorist operations, but also in major wars, for example in how different operations might affect allies. Equally, scientific advice cannot be politically innocent. It may seem necessary to protect the purity of the advice and to protect it from extraneous political influences, but an arms‐length relationship risks diminishing its value and impact. Getting the right questions and useful answers requires much closer engagement.

It is vital that experts maintain their independence and continue to offer conclusions, however awkward, based on their expertise. At the same time, it is the politicians who are responsible for what is done with the advice and, in the end, will be accountable to Parliament and the electorate. But for that reason, it is unwise for them to accept the expert advice too readily, confident that they can then claim that the resultant policies enjoy a certificate of scientific quality. At the very least, hard questions may provide some reassurance that the advice and its implications are understood. At best, the questions can force the experts to check their assumptions and acknowledge options that they might have been inclined to disregard. None of this requires policy makers to pretend to be more expert than the experts.

Much of the early analysis surrounding the coronavirus depended on the developing understanding on such questions as its infectivity, onset of symptoms, human impact and clinical demands. All this fed into the modelling that was central to the advice given to government on the likely scale and timing of the epidemic. The modellers tried to assess the impact of different interventions on its course. This is where political assessments came in, for example on the extent and sustainability of compliance, which led to arguments for a graduated response. But there was also a reason for the delay, which reflected a scientific inclination to wait for the evidence before making a big move. As better information came in from Italy, and from UK cases, there could be more confidence in what the modelling showed. With the Imperial College report, which became available to SAGE on 13 March and was published three days later, the message suddenly became urgent, because the disease was advancing at pace.^27^ The push for lockdown began. The government accepted that it had no choice but to take a major economic hit which will linger long after the health effects are receding.

The potential economic impact explains why the politicians were content to receive advice that allowed them more time in working out how best to ‘flatten the curve’. Could they have done more? It would be interesting to know what if any discussions took place between SAGE and the government on the validity of what was being assumed about the readiness of people to make sacrifices for the collective good. While the scientists might want to wait for the empirical evidence, the politicians did not need to do so. They should have been aware that over the first two weeks of March, especially in the second, the public mood was shifting. This was the result of a fear of infection and awareness of what was being done in other countries. Picking up on this sort of change in public attitudes and behaviour is an essential political skill. There was enough evidence of the gathering epidemic and the actions being taken by other European governments to underline the likelihood that tough measures would be adopted. This was, incidentally, the view of SAGE’s behavioural science group who observed on 13 March ‘that trust will be lost in sections of the public if measures witnessed in other countries are not adopted in the UK and that not pursuing such routes needs to be well explained’.^28^ There were also limits to the advice that SAGE could offer on operational matters such as testing, protective equipment, and respirators, or the need to check on care homes. By and large, at least early on in their deliberations, they assumed that these issues were being addressed. More intensive engagement might have highlighted the importance of this assumption and the need to check with the relevant bodies—NHS, Public Health England, Care Quality Commission—that appropriate measures were in place.

The problem, therefore, was not one of the political logic being at odds with scientific logic. The problem was that the coincidence of the political and scientific logics led to complacency. In these circumstances, when so much so much was at stake and the policy choices all carried risks, the need was for a persistent and intense engagement between the scientists and the policy makers. In such circumstances scientists must be sure that they are answering the right questions and that governments are aware of the available options. The conversation was important because even 'policy‐aware' scientists cannot anticipate all the questions or the way that their answers might be received.

The experience of the early pandemic response, along with that of Iraq, exposes the limitations of a model in which a specialist committee produces consensus statements that spare policy makers any requirement to make choices on matters in which they have no competence. These limitations paradoxically are going to be greatest when the advice is welcome and congenial, because when the policy consequences are controversial there is much more likely to be a degree of challenge and push back. A better model, to which the UK may now be tending because of the demands of Covid‐19, is a more integrated approach, with more opportunities to engage with the experts as both the advice and the policy is developed. There are obvious risks in politicising scientific advice, of the sort that were feared when it was reported that Dominic Cummings had attended SAGE meetings. The independence and integrity of the advice always needed to be safeguarded, even though when they are in conflict, politics is always likely to trump science. The transparency that now surrounds SAGE does provide one welcome form of protection: if the advice has been wilfully disregarded, this will be evident. But keeping the policy makers away from the experts until they are ready with an agreed position in order to ensure that their advice is not contaminated can come with heavy costs. This will be especially at times of emergency and uncertainty, when there is a degree of urgency, and the best evidence is not yet available.

## Notes

^1^
P. Vallance, ‘We are learning a lot, including how to do it better next time’, *Sunday Telegraph*, 31 May 2020; https://www.telegraph.co.uk/news/2020/05/30/will‐have‐learned‐lot‐including‐do‐better‐next‐time‐science/ (accessed 4 July 2020).

^2^
S. Carrell et al., ‘Revealed: Cummings is on secret scientific advisory group for Covid‐19’, *The Guardian*, 25 April 2020; https://www.theguardian.com/world/2020/apr/24/revealed‐dominic‐cummings‐on‐secret‐scientific‐advisory‐group‐for‐covid‐19 (accessed 4 July 2020).

^3^
The Independent Scientific Advisory Group for Emergencies (SAGE), *The Independent SAGE Report*. *COVID‐19: What are the Options for the UK? Recommendations for Government Based on an Open and Transparent Examination of the Scientific Evidence*, 12 May 2020; https://www.the‐eps.org/wp‐content/uploads/The‐Independent‐SAGE‐Report.pdf (accessed 4 July 2020).

^4^
GOV.UK, Scientific Advisory Group for Emergencies (SAGE): Coronavirus (COVID‐19) response. All materials added on a regular basis; https://www.gov.uk/government/groups/scientific‐advisory‐group‐for‐emergencies‐sage‐coronavirus‐covid‐19‐response (accessed 4 July 2020).

^5^
I discuss the story told in the minutes in L. Freedman, ‘Where the science went wrong’, *New Statesman*, 7 June 2020; https://www.newstatesman.com/science‐tech/coronavirus/2020/06/where‐science‐went‐wrong (accessed 4 July 2020).

^6^
P. Wintour and M. White, ‘Campbell claims BBC lied over Iraq “dodgy dossier”’, *The Guardian*, 26 June 2003; https://www.theguardian.com/media/2003/jun/26/broadcasting.politicsandiraq (accessed 4 July 2020).

^7^
GOV.UK, Independent report, *The Report of the Iraq Inquiry*, 6 July 2016, (Intelligence assessments are covered in Volume IV); https://www.gov.uk/government/publications/the‐report‐of‐the‐iraq‐inquiry (accessed 4 July 2020).

^8^
CIG Assessment, ‘The Status of Iraqi WMD Programmes’, 15 March 2002; https://webarchive.nationalarchives.gov.uk/20171123122951/http://www.iraqinquiry.org.uk/media/242501/2002‐03‐15‐cig‐assessment‐the‐status‐of‐iraqi‐wmd‐programmes.pdf (accessed 4 July 2020).

^9^

*Report of the Iraq Inquiry*, Vol. IV, pp. 293–5.

^10^

*Iraq’s Weapons of Mass Destruction. The Assessment of the British Government*, Cm. 5972, 24 September 2002, p. 3; https://fas.org/nuke/guide/iraq/iraqdossier.pdf (accessed 4 July 2020).

^11^

*Report of the Iraq Inquiry*, Vol. IV, pp. 372–5.

^12^
Ibid., pp. 344–5.

^13^
The development of military options for an invasion of Iraq is discussed in Volume V of the *Report of the Iraq Inquiry*.

^14^
Manuscript annotation by D. Manning on letter from Davies to Wechsberg, 12 August 2002, *Report of the Iraq Inquiry*, Vol. VI, p. 209.

^15^
GOV.UK, *The Permanent Joint Headquarters*; https://www.gov.uk/government/groups/the‐permanent‐joint‐headquarters (accessed 4 July 2020).

^16^
Ministry of Defence, *The Good Operation: A Handbook for those Involved in Operational Policy and its Implementation*, January 2018; https://assets.publishing.service.gov.uk/government/uploads/system/uploads/attachment_data/file/674545/TheGoodOperation_WEB.PDF (accessed 4 July 2020).

^17^
COBR simply stands for Cabinet Office Briefing Room but is now used to refer to the body convened at Cabinet level to address emergencies (and also known as Cobra).

^18^
House of Commons, Science and Technology Committee, Oral evidence from Professor Neil Ferguson to Q830, 10 June 2020; https://committees.parliament.uk/oralevidence/539/pdf/ (accessed 4 July 2020).

^19^
Ibid., Q845

^20^
GOV.UK, Addendum to eleventh SAGE meeting on Covid‐19, 27 February 2020; https://assets.publishing.service.gov.uk/government/uploads/system/uploads/attachment_data/file/888778/S0379_Eleventh_SAGE_meeting_on_Wuhan_Coronavirus__Covid‐19__.pdf (accessed 4 July 2020).

^21^
If R is above 1 the infection is spreading; if it is below it is in decline.

^22^
GOV.UK, SAGE 10 minutes: Coronavirus (COVID‐19) response, 25 February 2020; https://www.gov.uk/government/publications/sage‐minutes‐coronavirus‐covid‐19‐response‐25‐february‐2020 (accessed 4 July 2020).

^23^
Addendum to eleventh SAGE meeting.

^24^
Addendum to thirteenth SAGE meeting on Covid‐19, 5 March 2020; https://assets.publishing.service.gov.uk/government/uploads/system/uploads/attachment_data/file/888781/S0381_Thirteenth_meeting_on_Wuhan_Coronavirus__Covid‐19__.pdf (accessed 4 July 2020).

^25^
GOV.UK, ‘Potential impact of behavioural and social interventions on a Covid‐19 epidemic in the UK’, updated paper for the Scientific Advisory Group for Emergencies (SAGE), 9 March 2020; https://www.gov.uk/government/publications/potential‐impact‐of‐behavioural‐and‐social‐interventions‐on‐an‐epidemic‐of‐covid‐19‐in‐the‐uk‐9‐march‐2020 (accessed 4 July 2020).

^26^
GOV.UK, SAGE 14 minutes: Coronavirus (COVID‐19) response, 10 March 2020; https://assets.publishing.service.gov.uk/government/uploads/system/uploads/attachment_data/file/888782/S0382_Fourteenth_SAGE_meeting_on_Wuhan_Coronavirus__Covid‐19__.pdf (accessed 4 July 2020).

^27^
Imperial College COVID‐19 Response Team, *Report 9: Impact of Non‐pharmaceutical Interventions (NPIs) to reduce COVID‐19 Mortality and Healthcare Demand*, 16 March 2020; https://www.imperial.ac.uk/media/imperial‐college/medicine/sph/ide/gida‐fellowships/Imperial‐College‐COVID19‐NPI‐modelling‐16‐03‐2020.pdf (accessed 4 July 2020).

^28^
GOV.UK, ‘SPI‐B insights on public gatherings’, 12 March 2020, (addendum 13 March); https://www.gov.uk/government/publications/spi‐b‐insights‐on‐public‐gatherings‐12‐march‐2020 (accessed 4 July 2020).

